# Photoreforming Hydrogen From Polystyrene, Low‐Density Polyethylene, and High‐Density Polyethylene Microplastics via UV‐Driven Photolysis and TiO_2_‐Based Photocatalysis

**DOI:** 10.1002/cphc.70502

**Published:** 2026-07-26

**Authors:** Miroslava Filip Edelmannová, Petr Praus, Lenka Řeháčková, Rudolf Ricka, Michal Ritz, Kamila Kočí

**Affiliations:** ^1^ Institute of Environmental Technology CEET VSB‐Technical University of Ostrava Ostrava‐Poruba Czech Republic; ^2^ Department of Chemistry Faculty of Science University of Ostrava Ostrava Czech Republic; ^3^ Faculty of Materials Science and Technology VSB‐Technical University of Ostrava Ostrava‐Poruba Czech Republic

**Keywords:** HDPE, hydrogen production, LDPE, microplastics, photocatalysis, photoreforming, PS, TiO_2_

## Abstract

This study investigated UV‐induced photolysis and photocatalysis in the presence of TiO_2_ (Evonik P25) as methods for producing hydrogen from polystyrene (PS), low‐density polyethylene (LDPE), and high‐density polyethylene (HDPE) microplastics. Photolysis showed a strong dependence of reactivity on polymer structure, especially the one‐type LDPE microplastics, exhibiting the highest hydrogen production, while PS showed the lowest activity due to the stability of its aromatic backbone. Although thermodynamic analysis showed the lower Gibbs energy of decomposition for polyethylenes than for PS, all processes remained nonspontaneous. Photocatalysis selectively increased hydrogen production only in PS, where there was an increase of approximately 44% compared with photolysis, which is attributed to favorable polymer–photocatalyst interactions and more efficient charge transfer at the interface. In contrast for polyethylene microplastics, the presence of TiO_2_ led to a decrease in hydrogen yields, likely due to limited contact between the microplastics and the photocatalyst and radiation shielding by floating particles. Mixing proved to be a key operating parameter that significantly increases hydrogen production by improving particle dispersion and light distribution. Overall, the results show that the efficiency of microplastic photoreforming is governed by polymer chemistry, morphology, and system hydrodynamics under UV irradiation.

## Graphical abstract

UV‐driven photoreforming converts PS, LDPE, and HDPE microplastics into hydrogen, with efficiency governed by polymer structure and reaction conditions. TiO_2_ photocatalysis enhances hydrogen production for polystyrene but decreases performance for polyethylenes, where photolysis is more effective. Stirring improves particle dispersion and significantly increases hydrogen yield, highlighting the key role of system hydrodynamics.

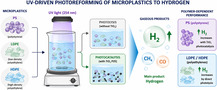

## 1 Introduction

Microplastics represent one of the most pressing environmental challenges of our time, as they accumulate in aquatic and terrestrial ecosystems and resist natural degradation processes. They are created by the fragmentation of larger plastic objects or are released directly into the environment as microparticles (e.g., from cosmetics, textiles). Due to their small size, large surface area, and chemical stability, microplastics have the ability to adsorb and transport toxic substances (metals, organic contaminants), posing a long‐term risk to organisms and humans. The most commonly detected polymers in the environment include PS and low‐density and high‐density polyethylene, reflecting their widespread industrial use and low biodegradability [1, 2, 3, 4, 5, 6, 7, 8, 9, 10, 11].

Traditional methods of plastic waste management, such as mechanical recycling, incineration, or physical separation, are proving to be ineffective for microplastics, mainly due to their dispersion, small size, and often high degree of contamination (e.g., the presence of additives). Recycling technologies are often unsuitable, and processes for removing microplastics from water systems are not yet fully integrated into real‐world wastewater treatment plants (WWTPs); moreover, most technological approaches are still in the laboratory phase [2, 3, 12].

Photocatalysis appears to be a promising way to simultaneously degrade microplastics and produce clean energy in the form of hydrogen. Semiconductor materials, especially titanium dioxide, generate electron–hole pairs when absorbing UV light, which create reactive species (e.g., hydroxyl radicals •OH, superoxide ions •O_2_^−^) capable of attacking polymer chains and breaking C—C and C—H bonds. This process can lead to photoreforming, where the organic structure acts as an electron donor for hydrogen evolution, creating a synergistic approach: plastic degradation with hydrogen production. The “plastic as a source of hydrogen” approach is discussed in review articles on the photoreforming of microplastics [1, 7, 13, 14, 15, 16, 17, 18, 19, 20, 21]. Although a variety of advanced photocatalysts, including metal–organic frameworks (MOFs) and covalent organic frameworks (COFs), have recently demonstrated promising performance for photocatalytic hydrogen production and solar fuel generation [22, 23], commercial TiO_2_ (Evonik P25) remains an important benchmark material owing to its high stability, low cost, commercial availability, and extensive use in photocatalytic studies. Therefore, it was selected in this work to evaluate the intrinsic influence of microplastic type on photoreforming performance using a well‐established reference photocatalyst.

Multiple experiments have shown that TiO_2_ can lead to almost the complete mineralization of some microplastics [24, 25]. For example, in one study, 400‐nm PS particles achieved the mineralization of up to ∼98.4 % within 12 h of UV irradiation on a TiO_2_ film (with Triton X‐100 added) [26]. For polyethylene, a high degree of photodegradation was observed within 36 h, with CO_2_ as the main end product [26]. Other studies show that the morphology of microplastics (size, shape) significantly affects the rate of degradation—smaller particles decompose faster than larger ones [16, 27]. In experiments with modified TiO_2_ (e.g., N doping), the photocatalytic degradation of LDPE and HDPE was investigated as a function of particle size, with results showing higher degradation for smaller particles [28].

This study investigates the UV‐induced degradation of PS, LDPE, and HDPE microplastics under both direct photolysis and photocatalytic conditions using commercial TiO_2_ (Evonik P25). The work focuses on the formation of gaseous products, particularly hydrogen, and on the influence of polymer structure and reaction conditions on degradation efficiency. By comparing photolysis and photocatalysis, this study aims to clarify when the presence of a photocatalyst enhances microplastic conversion and when direct UV irradiation remains more effective.

## 2 Experimental Section

### 2.1 Characterization of TiO_2_–P25 Photocatalyst

The commercially available TiO_2_ photocatalyst P25 was comprehensively characterized using X‐ray diffraction (XRD), Raman spectroscopy, N_2_ physisorption, UV–vis diffuse reflectance spectroscopy (UV–vis DRS), and scanning electron microscope (SEM). A detailed description of the performed characterization techniques is provided in the Supplementary Materials.

### 2.2 Preparation of Microplastics

The polymer granulates of PS, HDPE, and LDPE were ground in a cryogenic mill CryoMill (Retsch GmbH, Haan, Germany), and granulometry was adjusted to <0.16 mm using an analytical sieve shaker AS200 by Retsch (Haan, Germany) and a sieve by Preciselekt (Dolní Loučky, Czech Republic). PS granulates (No. 430102, MW = 192,000) were obtained from Sigma–Aldrich (Darmstadt, Germany). HDPE granulates (Hostalen GC 7260) were supplied by LyondellBasell Industries (Bayreuth, Germany). LDPE I granulates (Bralen+ FC 4–32) were obtained from Slovnaft (Bratislava, Slovak Republic), and LDPE II granulates were supplied by Roliband Embalagens, Lda (Portugal).

### 2.3 Characterization of Microplastics

The microplastics were characterized using advanced analytical methods, such as Fourier‐transform infrared (FTIR) spectrometry, elemental analysis, density measurements, and SEM. A detailed description of the performed characterization methods is given in the Supplementary Materials.

### 2.4 Photocatalytic Experiments

Photocatalytic experiments were performed in a closed batch photoreactor with an internal volume of 348 mL (Figure S1). A detailed description of the performed CO_2_ photoreduction experiments is provided in the Supplementary Materials.

## 3 Results and Discussion

### 3.1 Characterization of TiO_2_–P25 Photocatalyst

Commercially available TiO_2_ photocatalyst P25 was comprehensively characterized to determine its physicochemical properties and evaluate their influence on the photoreforming process. As shown in Figure S2a,b, XRD and Raman spectroscopy confirmed the expected mixed anatase/rutile phase composition together with the high crystallinity of the photocatalyst (Table 1). In addition, N_2_ physisorption analysis revealed a specific surface area of 54 m^2^.g^−1^ (Table 1), while the measured adsorption–desorption isotherm indicated the mesoporous character of P25, corresponding to a Type IV isotherm according to the IUPAC classification (Figure S2c). Further, UV–vis DRS and performed calculation of bandgap energy via Tauc plot demonstrated strong absorption of irradiation in the UV region with a determined bandgap energy of 3.2 eV (S2d–e, Table 1).

**TABLE 1:** Phase composition, crystallite size, specific surface area (*S*_BET_), and bandgap energy (*E*_g_) of TiO_2_ photocatalyst P25.

Sample	Anatase, %	Rutile, %	Anatase crystallite size, nm	Rutile crystallite size, nm	S_BET_, m^2^.g^−1^	*E*_g_, eV
TiO_2_–P25	91	9	30	37	54	3.2

In addition, SEM analysis was performed to evaluate the morphology of the commercially available TiO_2_ photocatalyst P25. As shown in Figure S3a–c, the SEM images revealed strongly agglomerated nanoparticles with irregular morphology. The agglomerates ranged in size from the submicrometer scale to tens of micrometers and are composed of spherical to slightly irregular primary particles. The observed morphology is consistent with the typical characteristics of commercial TiO_2_ P25, which consists of nanoparticles with dimensions in the range of approximately 20–30 nm.

### 3.2 Thermodynamic Analysis

To establish the thermodynamic driving force behind the photoreforming of representative microplastics, the overall oxidation pathways of PS (1) and polyethylene (2) were derived as follows:

(1) $$C_{8}H_{8\;}\left(s\right)+16\;H_{2}O\;(l)=8\;{\mathrm{CO}}_{2}\;\left(g\right)+20\;H_{2}\;(g)$$

(2) $$C_{2}H_{4\;}\left(s\right)+4\;H_{2}O\;(l)=2\;{\mathrm{CO}}_{2}\;(g)+6\;H_{2}\;(g)$$

These values were subsequently used for the evaluation of the standard Gibbs energy change:

(3) $$\Delta_{r}G_{298}^{0}=\Delta_{r}H_{298}^{0}-T\Delta_{r}S_{298}^{0}$$

Experimentally determined combustion and calculated formation enthalpies of HDPE, LDPE, and PS are in Table 2.

**TABLE 2:** Experimentally determined combustion and calculated enthalpies of investigated plastics.

Plastics	Δ$H_{298,\mathrm{comb}}^{0}$, kJ·mol^−1^	Δ$H_{298,f}^{0}$, kJ·mol^−1^
PS	−4307	191.3
HDPE	−1266	−4.7
LDPE I	−1302	31.2
LDPE II	−1301	29.3

The combustion reactions of PS and PE can be described by reactions:

(4) $$C_{8}H_{8\;}\left(s\right)+10\;O_{2}\;\left(g\right)\to 8\;{\mathrm{CO}}_{2}\;(g)+4\;H_{2}O\;(g)$$

(5) $$C_{2}H_{4\;}\left(s\right)+3\;O_{2}\;\left(g\right)\to 2\;{\mathrm{CO}}_{2}\;(g)+2\;H_{2}O\;(g)$$

The enthalpies of these reactions can be calculated according to the Hess's law using the data given in Table 3:

**TABLE 3:** Standard enthalpies and entropies used for calculations.

Substance	Δ$H_{298,f}^{0}$, **kJ·mol** ^−^ **^1^**	Δ$S_{298}^{0}$, **kJ·K** ^−1^·mol^−1^
H_2_ (g)	0	130.68
O_2_ (g)	0	205.15
H_2_O (g)	−241.83	188.84
H_2_O (L)	−285.83	69.95
CO_2_ (g)	−393.52	213.79

(6) $$\Delta_{r}H_{298}^{0}=\Delta H_{298,\mathrm{comb}}^{0}=\sum_{i}{\left(\upsilon_{i}\Delta H_{f,i}^{0}\right)}_{\text{product}}-\sum_{i}{\left(\upsilon_{i}\Delta H_{f,i}^{0}\right)}_{\text{reactant}}$$

The reaction entropies of the Reactions (4) and (5) were calculated as follows using the data in Table 3:

(7) $$\Delta_{r}S_{298}^{0}=\sum_{i}{\left(\upsilon_{i}\Delta S_{i}^{0}\right)}_{\text{product}}-\sum_{i}{\left(\upsilon_{i}\Delta S_{i}^{0}\right)}_{\text{reactant}}$$

The calculated thermodynamic parameters summarized in Table 4 provide important information about the feasibility of the photoreforming reactions of microplastics. For all polymers studied, the reaction enthalpies (Δ*_r_* *H* ^0^_298_) are positive, indicating that the overall oxidation of polyethylene and PS associated with hydrogen formation is endothermic. This behavior is expected because the degradation of polymer backbone chains requires a significant supply of energy.

**TABLE 4:** Calculated reaction enthalpies, entropies, and the Gibbs energies for Reactions (1) and (2) .

Plastics	Δ_r_ $H_{298}^{0}$, kJ·mol^−1^	Δ_r_ $S_{298}^{0}$, kJ·K^−1^·mol^−1^	Δ_r_ $G_{298}^{0}$, kJ·mol^−1^
PS	1233.9	3.19	283.3
HDPE	360.9	0.92	86.1
LDPE I	325.1	0.92	50.2
LDPE II	327.0	0.92	52.1

Despite the endothermic nature of the reactions, the calculated Gibbs reaction energies (Δ*_r_* *G* ^0^_298_) for polyethylene‐based polymers (HDPE and LDPE) are significantly lower than for conventional water splitting (2H_2_O → O_2_ + 2H_2_), which requires 237 kJ·mol^−1^. For polyethylene in particular, Δ*_r_* *G* ^0^_298_ values in the range of 50–86 kJ·mol^−1^ were obtained, indicating a significantly reduced thermodynamic barrier for hydrogen production when polymers act as sacrificial electron donors. In contrast, PS exhibits a significantly higher Δ*_r_* *G* ^0^_298_ value (283.3 kJ·mol^−1^), which exceeds the value for water splitting and reflects the higher thermodynamic stability of its aromatic chain.

The differences in the Gibbs energies between polymers can be attributed to their molecular structure and elemental composition. Polyethylene, composed of saturated aliphatic chains with a high H/C ratio (Table 5), thermodynamically favors hydrogen evolution, while the aromatic structure of PS stabilizes the carbon backbone and increases the energy cost of oxidation. These findings are consistent with the calorimetric data and elemental analysis presented above and confirm that polymer composition plays a key role in determining the thermodynamic driving force of photoreforming.

**TABLE 5:** Elementary composition, combustion heats, and density of investigated plastics.

Plastics	Elemental analysis			Calorimetry	Density, g·cm^−3^
	C, wt.%	H, wt.%	N, wt.%	Combustion heat, kJ·kg^−1^	
PS	92.08	7.79	0.13	41,411	1.10
HDPE	85.47	14.37	0.16	46,495	0.96
LDPE I	85.54	14.27	0.19	45,216	0.95
LDPE II	88.84	14.16	<0.05	46,475	0.92

*Note:* The error of determination was 5%.

Importantly, although the Gibbs energies of reaction for polyethylene are lower than or comparable to the energy of water splitting, all the calculated Δ*_r_* *G* ^0^_298_ values remain positive. This suggests that the reactions are not spontaneous under standard conditions and require an external energy input to proceed. Therefore, photons and an active photocatalyst are necessary to overcome the remaining thermodynamic barrier and to carry out these reactions. From a practical point of view, this highlights the role of microplastics not as the independent sources of energy but as thermodynamically advantageous sacrificial agents that can significantly reduce the energy intensity of photocatalytic hydrogen production compared with the decomposition of pure water.

The thermodynamic analysis confirms that, except for PS, the oxidation of polymers is more energy efficient than conventional water splitting, especially for polyethylenes, while still requiring photocatalytic activation. These results provide a solid thermodynamic rationale for experimental research into photocatalytic hydrogen production from microplastics.

### 3.3 FTIR Spectroscopy

**FIGURE 1:**
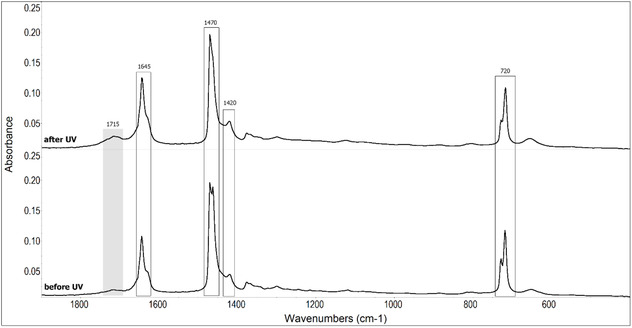
IR spectra of bulk samples of LDPE II before UV and after UV exposure.

The FTIR spectra of microplastics (before and after UV exposure) showed no spectral changes (Figure 1 and Figures S4–S6). From the perspective of FTIR spectroscopy, no alterations were observed in the samples following UV exposure.

However, in the spectra of the LDPE II microplastics before and after UV exposure, a slight change was noticeable. In Figure 1, this change is visible in the region around 1700 cm^−1^, which is characteristic of the stretching vibrations of the carboxyl functional group (C=O). In the spectrum of the sample before UV exposure, no spectral band is visible in this region, whereas in the spectrum of the sample after UV exposure, a weak band at 1715 cm^−1^ is clearly present.

It can therefore be assumed that UV radiation induced an interaction between oxygen in water and the microplastics in the aqueous suspensions, resulting in the incorporation of carboxyl groups into the chemical structure of the microplastics.

Other spectral bands observed in FTIR spectra in Figure 1 belong to various vibrational modes. The bands at 1645 cm^−1^ can be attributed to the valence vibration of C=C in aliphatic hydrocarbons. The deformation vibrations can be observed at 1470 cm^−1^ of C–H in aliphatic hydrocarbon (CH_2_ group; asymmetric mode), at 1420 cm^−1^ of C–H aliphatic hydrocarbon (CH_2_ group; symmetric mode—so called “scissoring” mode), and at 720 cm^−1^ of deformation (“rocking”) vibration of CH_2_ group (coupled vibration of CH_2_ groups in aliphatic hydrocarbon chains).

### 3.4 Elemental Composition and Physicochemical Properties

The elemental composition of the investigated microplastics (Table 5) reflects their predominantly hydrocarbon nature, with carbon and hydrogen as the major constituents and only trace amounts of nitrogen. Polystyrene (PS) shows the highest carbon content (92.08 wt%), which is consistent with its aromatic backbone. In contrast, polyethylenes (HDPE, LDPE I, and LDPE II) exhibit slightly lower carbon contents (∼85–86 wt%) and higher hydrogen fractions (∼14 wt%), as expected for saturated aliphatic polymer backbones.

These compositional differences are reflected in the calorimetric data. Among the tested microplastics, HDPE and LDPE exhibit the highest combustion heats (≈45.2–46.5 MJ·kg^−1^), which can be attributed to their high hydrogen content and fully saturated hydrocarbon structure. PS shows a lower heat of combustion (41.4 MJ·kg^−1^), in agreement with the presence of aromatic rings, which generally reduces the specific energy requirements compared with fully aliphatic polymers.

Density measurements further support the structural differences between the materials. PS exhibits the highest density (1.098 g·cm^−3^) as a consequence of its rigid aromatic structure, while polyethylenes remain significantly less dense (0.92–0.96 g·cm^−3^), which is consistent with their semicrystalline aliphatic character (crystallinity of 45%–55%) [29]. The modified LDPE II sample shows the lowest density (0.92 g·cm^−3^), which may indicate subtle structural or morphological changes.

The observed trends in elemental compositions, calorific values, and densities align well with the known structural characteristics of PS and polyethylene‐based materials, providing a consistent basis for interpreting their subsequent thermal and combustion behavior.

### 3.5 SEM

**FIGURE 2:**
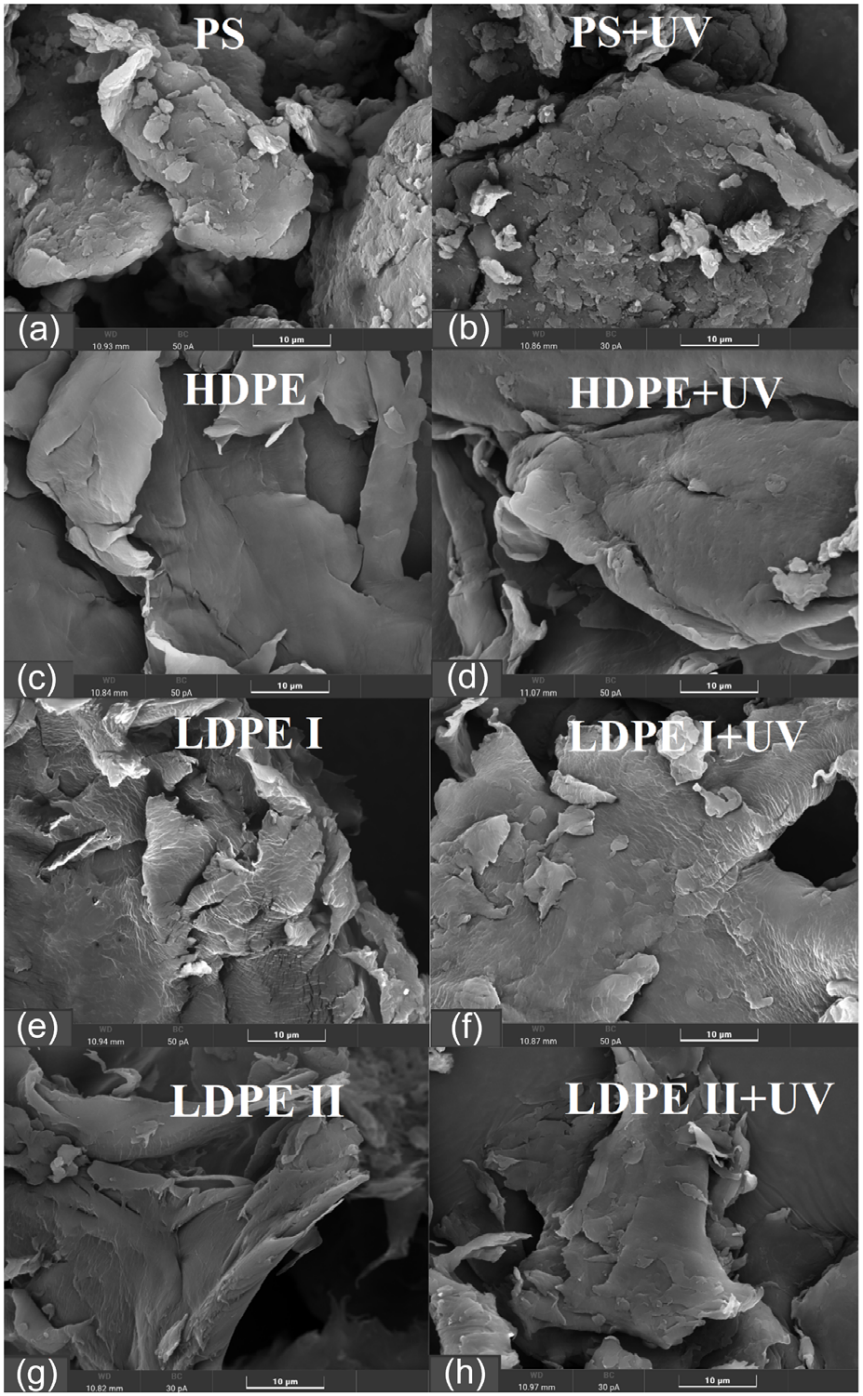
SEM images of investigated microplastics; (a), (c), (e), and (g) before photolysis reaction and (b), (d), (f), and (h) after 4 h of UV irradiation.

Scanning electron microscopy (SEM) was used to examine morphological changes in microplastic surfaces before and after UV irradiation (Figure 2) and to evaluate the effect of photodegradation on surface integrity. Before irradiation, all intact microplastics exhibited smooth, compact, and homogeneous surfaces characteristic of untreated plastic materials. Polyethylene‐based particles exhibited a uniform morphology with low surface roughness, consistent with their semicrystalline structure, while the PS particles exhibited a denser and stiffer surface structure.

After UV irradiation, significant morphological changes were observed in all the microplastics examined. All samples showed surface roughening, microcrack formation, and increased surface fragmentation, indicating structural degradation. The most significant changes were observed in HDPE, which showed the delamination and erosion of sharp surface features. LDPE I developed a wrinkled and flaky morphology, accompanied by signs associated with surface oxidation. Similarly, LDPE II exhibited pronounced degradation of the polymer matrix after UV exposure, characterized by a markedly roughened and cracked surface with the presence of microcracks and fragmented regions, consistent with UV‐induced oxidative degradation and polymer chain scission. PS also showed degradation effects, manifested by a loss of surface compactness and the formation of fine granular structures on the surface, probably related to the degradation of aromatic structure. UV irradiation led to a significant deterioration in the surface morphology of the microplastics, with the extent of degradation depending on the type of its polymer backbone and its internal structural characteristics.

### 3.6 Photoreforming of Microplastics

The photoreforming of microplastics of PS, HDPE, and LDPE was performed under UV radiation with a wavelength of 254 nm. Three main gaseous products were identified: hydrogen, methane, and carbon monoxide. At the beginning of photoreforming experiments, the stirring of microplastic suspensions was optimized.

#### 3.6.1 Stirring Effect

**FIGURE 3:**
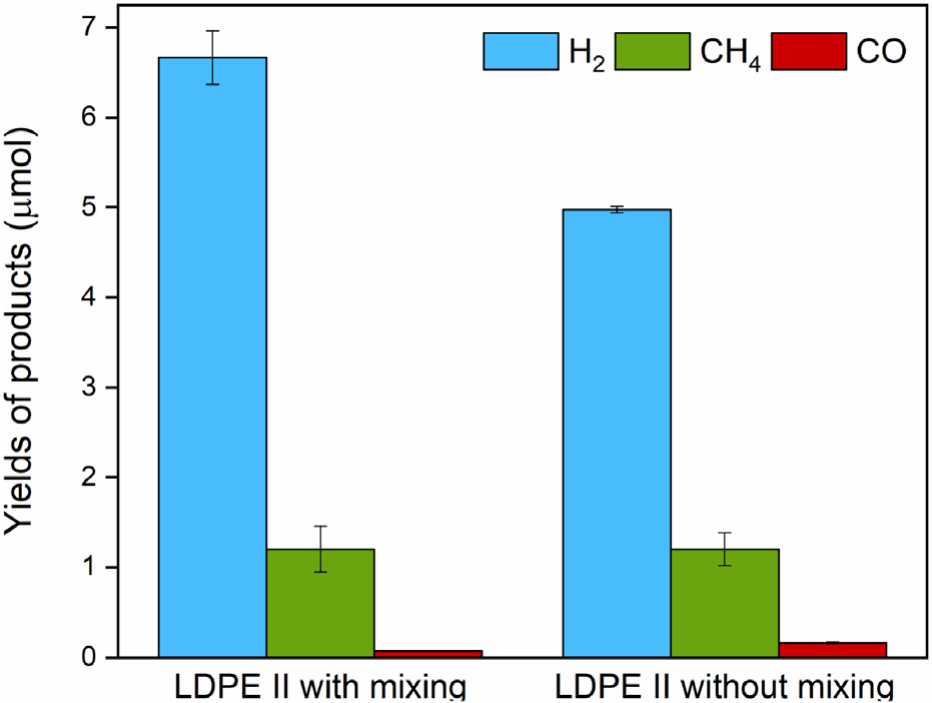
Comparison of photocatalytic results in the presence of LDPE II with and without stirring.

To assess the effect of stirring on the photoreforming conversion of microplastics, photolytic experiments were conducted using LDPE II. The formation of gaseous products was monitored under stirring conditions (350 rpm) and without stirring. The results (Figure 3) clearly show that stirring has a significant positive effect on photocatalytic hydrogen production. Under stirring conditions, a hydrogen yield of 6.66 µmol was achieved, while, without stirring, only 4.98 µmol was produced, corresponding to an increase of approximately 34%. In contrast, methane production remained essentially unchanged (∼1.20 µmol), while carbon monoxide formation was higher in the unstirred system (0.161 µmol) compared with stirring (0.07 µmol). The observed effect can be attributed to several factors.

Stirring significantly improves the dispersion of LDPE II particles, as well as the photocatalyst within the reaction medium, thereby increasing the effective surface area exposed to UV radiation. At the same time, particles agglomeration is suppressed, leading to improved contact among the microplastics, water, and, in case of photocatalysis, also TiO_2_. Another important aspect is the more homogeneous distribution of light throughout the reaction volume, which promotes the more efficient formation of reactive radical species and consequently accelerates the degradation of polymer chains.

The lower CO production observed under mixing conditions suggests that the system favors the further reduction of reaction intermediates and exhibits higher selectivity toward hydrogen. In contrast, an unstirred system may suffer from local depletion of reactive species, leading to partial oxidation of carbon‐containing fragments. These findings confirm that mixing is a key operating parameter that significantly affects both the efficiency and selectivity of the photocatalytic conversion of microplastics into energy‐rich gaseous products.

Time‐dependent analysis of CO formation (Figure S7) revealed that the CO concentration initially increased during the first 2 h of irradiation and subsequently decreased with prolonged reaction time. This decrease was more pronounced under stirring conditions, indicating that mixing promotes the further transformation or consumption of CO and other carbon‐containing intermediates during the photoreforming process.

The influence of stirring is further evidenced by visual observations of microplastic distribution in the reaction medium under stirred and nonstirred conditions (Figure S8).

Based on these results, all further photoreforming experiments, both photolytic and photocatalytic, were performed under continuous stirring to ensure optimal reaction conditions.

#### 3.6.2 Photolysis of Microplastics

**FIGURE 4:**
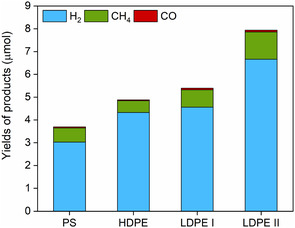
Yields of photolytic products after 4 h of irradiation in the presence of microplastics.

Photolytic experiments were conducted without the presence of a photocatalyst in order to assess the intrinsic reactivity of individual microplastics under UV irradiation. A graphical comparison of the yields of gaseous products (H_2_, CH_4_, and CO) after 4 h of irradiation is shown in Figure 4, and corresponding numerical values are summarized in Table S1. In all the cases, hydrogen was the main product, while methane and carbon monoxide occurred only in low concentrations.

Time‐dependent evolution of hydrogen during photolysis is shown in Supplementary Information (Figure S9), confirming that hydrogen is produced continuously throughout the irradiation period, with no significant stagnation observed during the first 4 h.

**FIGURE 5:**
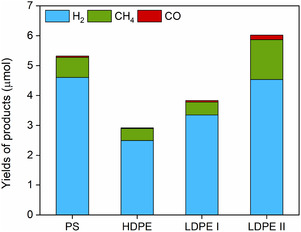
Yields of photocatalytic products after 4 h of irradiation in the presence of microplastics.

The total gas yields showed a clear trend of LDPE II > LDPE I ≈ HDPE > PS, as shown in Figure 5. The highest hydrogen production was achieved with the LDPE II sample, while PS showed the lowest activity. The consistently low CO concentrations observed for all the microplastics indicate that oxidative degradation of the polymer backbone was limited under the applied conditions. The degradation process is initiated by the breaking R—H bond forming R• and H• radicals [30], and then hydrogen radicals form hydrogen [31]. The oxidative degradation under UV irradiation can be described by the Norrish I and Norrish II mechanisms [30, 32]. These mechanisms also lead to the formation of methane and CO. Oxygen involved in these reactions was dissolved in water used for the photoreforming experiments. In addition, it should be noted that the UV absorption observed for the LDPE and HDPE microplastics can be attributed to the presence of additives in virgin plastics that are not disclosed by manufacturers.

Photolytic experiments after 4 h of irradiation showed significant differences in the reactivity of individual polymers. In all cases, hydrogen was the main product. The highest H_2_ production was recorded for the LDPE II sample (6.66 µmol), while PS showed the lowest activity (3.03 µmol).

The low reactivity of PS is related to its aromatic structure, which provides the polymer backbone with high stability against UV radiation and limits the formation of radical intermediates. In the case of HDPE, its photolytic activity is limited by a high degree of crystallinity 70%–80% [29], which reduces UV penetration and the availability of reactive sites in the material volume. In contrast, LDPE exhibits a more amorphous and branched structure that is more susceptible to photochemical cleavage. This is reflected in the higher production of gaseous products, especially hydrogen. The increased activity of the LDPE II sample may also be associated with a higher content of oxidative defects, which facilitate the initiation of photodegradation processes.

The observed differences are also consistent with the thermodynamics of the decomposition of individual polymers. The lower Gibbs energy of decomposition ($\Delta_{r}G_{298}^{0}$) of LDPE makes its degradation more energetically favorable and promotes higher gas yields. Conversely, the high $\Delta_{r}G_{298}^{0}$ value for PS corresponds to its low photolytic reactivity. The methane production remained low for all the microplastics (0.52–1.20 µmol), indicating a limited extent of cleavage.

#### 3.6.3 Photocatalysis of Microplastics

Photocatalytic experiments after 4 h of irradiation showed significant differences in the activity of individual microplastics (Table S1 and Figure 5). The time course of hydrogen production during photocatalysis is shown in Supporting Information (Figure S10), where the different kinetic behaviors of the individual polymers are evident and where it is confirmed that the values reported for 4 h reflect different reaction rates rather than merely differences in the final conversion. The total yields of gaseous products again followed the trend LDPE II > PS ≈ LDPE I > HDPE. In all cases, hydrogen was the dominant product, while methane and carbon monoxide were formed in smaller quantities. However, the presence of the photocatalyst led to different behavior of individual microplastics compared with photolysis.

PS showed significantly higher reactivity in photocatalysis despite its unfavorable thermodynamics of decomposition ($\Delta_{r}G_{298}^{0}$ = 283.3 kJ·mol^−1^). The H_2_ production reached 4.60 µmol, which is a significant improvement over photolysis. This effect can be attributed to interactions between the microplastics and the TiO_2_ surface. The aromatic structure of PS allows π–π interactions with the photocatalyst, which promotes polymer adsorption, facilitates charge transfer at the interface and increases the formation of reactive radicals. These interactions can partially overcome thermodynamic limitations and significantly increase the photocatalytic activity of PS.

In contrast, HDPE exhibited the lowest gas yields of all tested microplastics (2.49 µmol H_2_, 0.41 µmol CH_4_, and 0.02 µmol CO). The low activity is associated with a high degree of crystallinity, which limits the mobility of polymer chains and reduces the number of surface‐accessible reactive sites. The crystalline structure also prevents the effective interactions of the polymer with photogenerated charge carriers, resulting in less efficient electron and hole transfer at the polymer–photocatalyst interface.

LDPE‐based samples showed the best photocatalytic performance. In particular, LDPE II achieved the highest total gas yields (4.54 µmol H_2_, 1.33 µmol CH_4_, and 0.16 µmol CO). This increased activity is related to the amorphous and branched structure of LDPE and the presence of oxidative defects, which facilitate the activation of the polymer chain and the formation of radical intermediates. Higher structural disorder improves contact with the photocatalyst surface and allows for the more efficient utilization of photogenerated electrons and holes.

**FIGURE 6:**
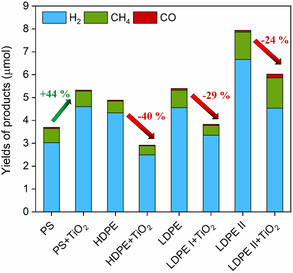
Comparison of photocatalysis and photolysis results in the presence of PS, HDPE, and LDPE microplastics.

A direct comparison of photolysis and photocatalysis shows that the effect of the photocatalyst is not only determined by the chemical structure of the polymers but is also significantly influenced by physical properties, in particular the density and behavior of particles in the reaction medium. As shown in Figure 6, the presence of TiO_2_ (P25) led to a significant increase in the total yield of gaseous products only in the case of PS, where an increase of approximately 44% was recorded.

The increased photocatalytic efficiency of PS is related not only to its aromatic structure, which enables π–π interactions with the TiO_2_ surface, but also to the higher density of the polymer. PS particles appears to exhibit more homogeneous dispersion in the reaction medium and remain in good contact with the photocatalyst throughout the experiment. This may facilitate effective polymer adsorption, charge transfer at the polymer–photocatalyst interface, and the formation of reactive radicals, which lead to higher gas product yields. In this case, photocatalytic interactions may partially overcome the unfavorable thermodynamics of PS decomposition. The different behavior of the microplastics in photocatalytic experiments is further supported by the visual observations of particle distribution in the reaction medium, which are provided in the Supporting Information (Figure S11).

In contrast, for HDPE and LDPE, the introduction of the photocatalyst led to a reduction in total gas yields. HDPE showed a decrease of approximately 40%, while LDPE I and LDPE II showed decreases of 29% and 24%, respectively. These polymers have a lower density than water and tend to float on the surface of the reaction medium, which significantly limits their contact with dispersed TiO_2_ particles. Limited interaction at the polymer–photocatalyst interface reduces the efficiency of photogenerated charge carrier transfer and suppresses photocatalytic reaction pathways.

In addition, the high crystallinity of HDPE and the structural character of LDPE further limit the availability of reactive sites and the effective activation of polymer chains. As a result, direct photolysis remains a more effective route than photocatalysis for these microplastics under the given conditions. Although LDPE II exhibits the highest overall reactivity of all the plastics studied, its susceptibility to photolytic degradation is not further enhanced by the presence of TiO_2_.

The stability of the photocatalytic system was further evaluated for the PS sample. As shown in Figure S12, four consecutive cycles were performed under identical conditions, confirming that no significant deactivation occurred during repeated use. Hydrogen production remained stable with a value of 4.60 ± 0.14 µmol after 4 h in each cycle.

In addition to hydrogen production, future development of such systems may involve coupling microplastic photoreforming with simultaneous CO_2_ conversion, enabling integrated waste utilization and solar fuel production, as recently suggested in related photocatalytic studies [33]. Such approaches could further enhance the sustainability and environmental relevance of photocatalytic plastic valorization.

## 4 Conclusion

This work has demonstrated that the photochemical conversion of microplastics is strongly dependent on the chemical structure, morphology, thermodynamic stability, and physical behavior of polymers in the reaction medium. Direct photolysis was the most effective for LDPE, especially for the LDPE II sample, while photocatalysis with TiO_2_ significantly increased the yields of gas products only for PS microplastics due to strong interface interactions and stable particle suspension. For polyethylenes, direct photolysis remained more effective than photocatalysis under the conditions used, which is related to their low density, resulting in limited contact with the photocatalyst, and structural barriers to charge transfer. The SEM analysis of microplastics, performed exclusively after UV irradiation without the presence of a photocatalyst, confirmed significant changes in the surface morphology of all studied polymers, including roughening, microcracks, and fragmentation. The varying extent of these changes between microplastic supports the conclusion that their structure fundamentally influences the mechanism and intensity of photodegradation. Overall, the results show that the effectiveness of photocatalysis cannot be generalized and its contribution is highly polymer specific.

## Funding

This work was supported by Ministerstvo Životního Prostředí (Grant CZ.10.03.01/ 00/22_003/0000048) and Ministerstvo Školství, Mládeže a Tělovýchovy (Grants CZ.02.01.01/00/23_021/0008588, LM2023056).

## Conflicts of Interest

The authors declare no conflicts of interest.

## Supporting information

Supplementary Material

## Data Availability

The data that support the findings of this study are openly available in ZENODO at https://doi.org/10.5281/zenodo.18267743.
