# Supplementary material for: Photoreforming Hydrogen From Polystyrene, Low‐Density Polyethylene, and High‐Density Polyethylene Microplastics via UV‐Driven Photolysis and TiO2‐Based Photocatalysis
Source: Chemphyschem. 2026 Jul 26;27(14):e70502. doi: 10.1002/cphc.70502 (PMC13402217; doi:10.1002/cphc.70502)
Supplement: Supplementary file 1 — Supplementary Material [file CPHC-27-e70502-s001.pdf]

Supplementary Materials

Photoreforming Hydrogen from PS, LDPE and HDPE Microplastics via UV-driven Photolysis and TiO_2-based Photocatalysis

Miroslava Filip Edelmanová)[a], Petr Praus[a][b], Lenka Řeháčková[c], Rudolf Ricka[a], Michal Ritz[c], Kamila Kočí*[a]

Dedication

[a] prof. Ing. Kamila Kočí, Ph.D. Ing. Miroslava Filip Edelmannová, Ph.D., pro. Ing. Petr Praus, Ph.D., Ing. Rudolf Ricka, Ph.D.
Institute of Environmental Technology
CEET, VSB-Technical University of Ostrava
17. listopadu 15, 708 00 Ostrava-Poruba, Czech Republic
E-mail: kamila.koci@vsb.cz

[b] prof. Ing. Petr Praus, Ph.D.
Department of Chemistry
Faculty of Science, University of Ostrava
30. dubna 22, Ostrava 701 03, Czech Republic

[c] doc. Ing. Lenka Řeháčková, Ph.D., doc. Ing. Michal Ritz, Ph.D.
Faculty of Materials Science and Technology
VSB-Technical University of Ostrava
17. listopadu 15, 708 00 Ostrava-Poruba, Czech Republic

Characterization of TiO_2 photocatalyst P25

X-ray diffraction (XRD, Rigaku SmartLab diffractometer, Rigaku, Tokyo, Japan) with detector D/teX Ultra 250 was used to analyze the phase composition and crystallite size of the TiO_2 photocatalyst P25. As the source of XRD irradiation, a Co tube (CoKα, λ_1 = 0.178892 nm, λ_2 = 0.179278 nm) operated at 40 kV and 40 mA was used.

The Raman spectra of the investigated TiO_2 – P25 was obtained by the dispersive Raman spectrometer DXR SmartRaman (ThermoScientific, USA), using a 532 nm excitation laser. The exposure time during this measurement was 1 s, and the number of repetitions per measurement was 1000.

The textural parameters of the TiO_2 photocatalyst P25 were determined by N_2-adsorption-desorption measurements. Measurement was carried out at a temperature of 77 K and using a QUADRASORB evoTM Gas Sorption analyzer (Anton Paar GMBH, Austria). Before the analyses, the material was degassed at 373 K for 16 h under a high vacuum using the MasterPrep sample degassing system (Anton Paar GmbH, Austria).

Optical properties and values of the band gap energies of the TiO_2 photocatalyst P25 were obtained using the V-650 UV-Vis spectrophotometer (JASCO International Co., Japan). For the examination of DR spectra, this device is equipped with a PIV-756 integrating sphere accessory (JASCO International Co., Japan). The barium sulphate ($BaSO_4$, pure p.a., Avantor Performance Materials Poland S.A.) was used as reference material.

The morphology of the TiO_2 photocatalyst P25 was analyzed using a scanning electron microscope (Tescan Vega) equipped with a tungsten cathode. Micrographs were acquired using a combination of secondary electron and backscattered electron signals (SE+BSE) at an accelerating voltage of 15 keV.

Characterization of microplastics

The Fourier Transform Infrared (FTIR) spectra of all powdered samples (microplastics) were collected by the Attenuated Total Reflection (ATR) technique. A single-reflection diamond crystal was used. The samples were not modified in any way prior to measurement; they were simply placed on the ATR crystal and pressed against it using a pressure clamp to ensure optimal contact between the sample and the crystal. The spectra were collected using a FTIR spectrometer Nicolet iS50 (Thermo Scientific, Madison, WI, USA) with a DTGS detector. The following parameters were used for measurement: a spectral region of 4000–400 cm^{-1}; a spectral resolution of 4 cm^{-1}; 512 scans; the Happ–Genzel apodization. The measured spectra were processed using ATR correction, and the baseline was also adjusted using a second-order polynomial function.

The density of the investigated microplastics was determined using a Pycnomatic ATC helium pycnometer (Porotec, Germany). Helium with a purity of 4.8 was used as the measuring gas.

The calorific value of microplastics was determined using an AC600 (LECO) calorimeter, which enables accurate measurement of the heat released during complete combustion of the sample in a controlled environment. The heat released is directly proportional to the calorific value of an analyzed substance. Individual analyses are performed with high reproducibility and the measurement time does not exceed 10 minutes per sample. A representative sample weighing ~1 g was prepared for measurement in accordance with ASTM D5865. The calorimeter was calibrated using standard benzoic acid pellets (LECO 774-208) or pellets prepared from the certified NIST 39j material. Combustion took place in a standard calorimetric bomb using a fuse or cotton wool.

The elemental analysis of microplastics was performed on a CHNS 628 analyzer (LECO, USA), which enables quantitative determination of carbon, hydrogen, and nitrogen based on complete oxidation of the sample in a stream of pure oxygen. Individual elements are detected using infrared cuvettes to determine the concentration of CO_2 and H_2O (corresponding to the C and H content) and thermal conductivity cuvettes to detect nitrogen. The sample was oxidized at a temperature of 950–1050 °C, followed by burnout of residual particles at 850 °C. A representative sample weighing ~100 mg was prepared for analysis and placed in tin crucibles (502-186). The instrument was calibrated using certified standards such as phenylalanine (502-642), EDTA (502-092), nicotinic acid (501-050), or other suitable pure compounds. Sucrose (501-441) was used as a reference material to check the accuracy of the measurements. The operation of the instrument required the use of helium, oxygen, and compressed air gases, which provided the carrier and combustion gases for the individual steps of the analysis.

The microscopic characterization of microplastics was performed using a scanning electron microscope (Tescan Vega) equipped with a tungsten cathode. Micrographs were obtained using a combination of secondary electron and backscattered electron signals (SE+BSE) at an acceleration voltage of 15 keV. Prior to imaging, the samples were gold-coated to ensure sufficient electrical conductivity. Images were taken from randomly selected areas of the samples to provide an unbiased view and to avoid focusing on local anomalies or artifacts.

Photocatalytic experiments

The reaction system consisted of 100 ml of an aqueous suspension containing 0.5 g of the investigated microplastics, supplemented with 100 ml of demineralized water and, where appropriate, 0.05 g of commercial TiO_2 (P25). Before irradiation, the reactor was hermetically sealed and flushed with helium to remove atmospheric oxygen.

Illumination was provided by a UV lamp with a wavelength of 254 nm, which was horizontally mounted above the reactor and directed through a quartz glass window. Before irradiation began, an initial sample of gas phase was taken through the partition using a gas-tight syringe. The composition of the gas products was then determined using a gas chromatograph (Shimadzu Nexis GC-2030) equipped with a dielectric barrier ionization detector (BID).

The photocatalytic reaction was carried out for 4 hours under continuous irradiation. Gas phase samples were taken after 1, 2, 3, and 4 hours and analyzed using GC-BID. All experiments were performed at least three times to verify reproducibility. Hydrogen, methane, and carbon monoxide were consistently determined as the main gas products under these photocatalytic conditions. The stability of the photocatalyst and microplastics was evaluated by reusing the same batch of photocatalyst and microplastics in at least three consecutive experiments, with no significant changes in product distribution or reaction performance observed.

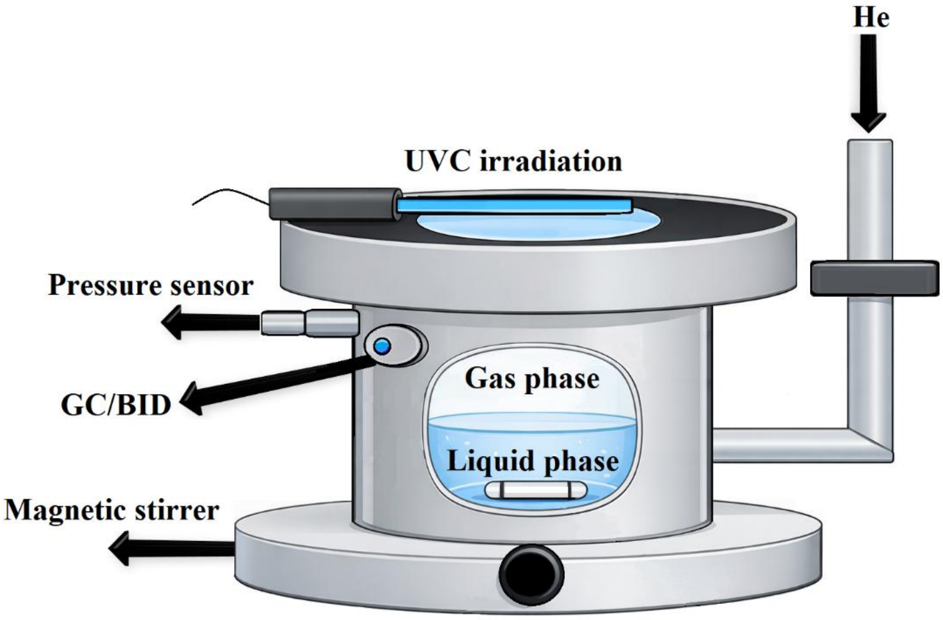

Figure S1: The schematic illustration of the used photocatalytic reactor.

Results

Results of characterization of P25

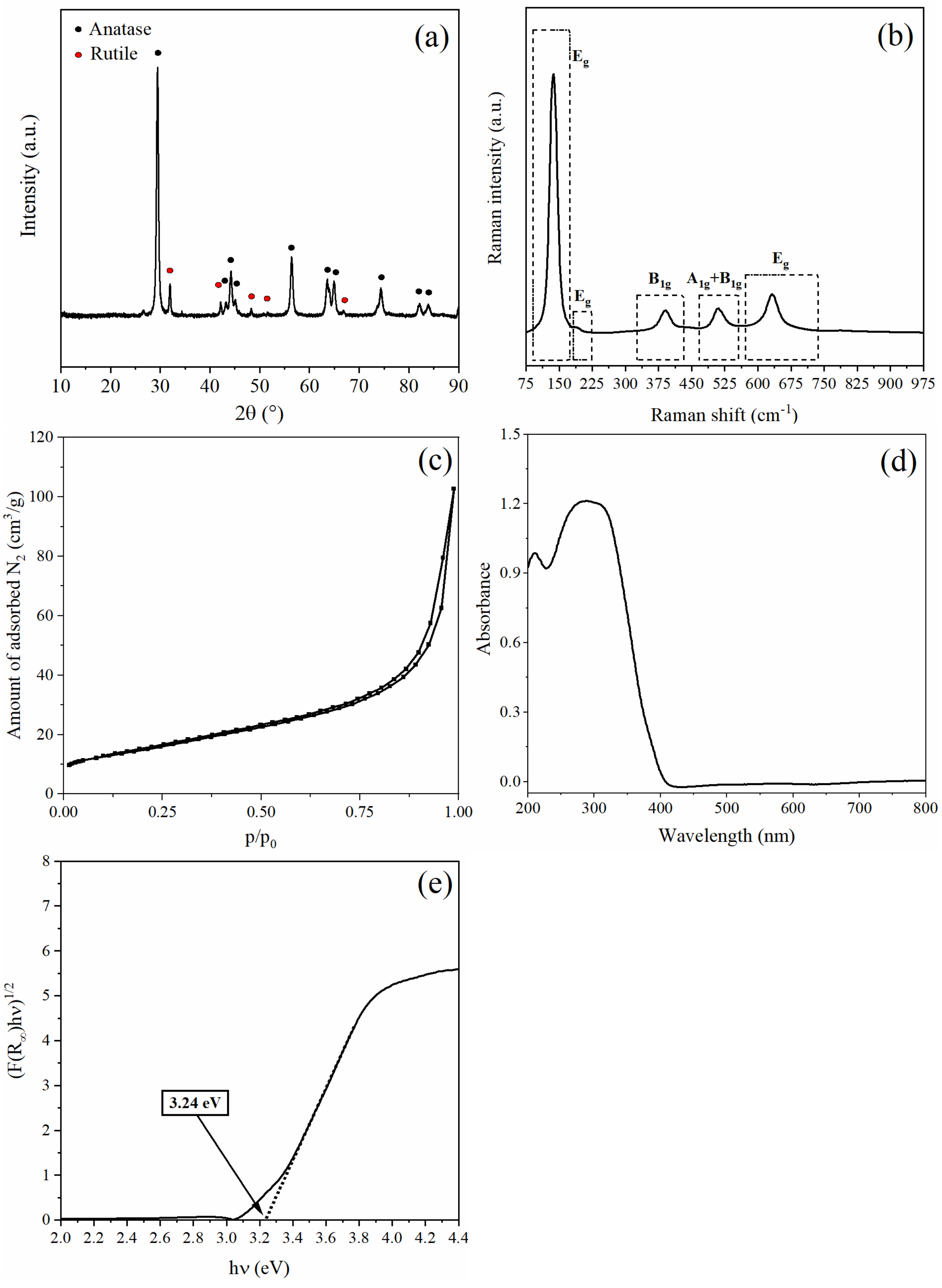

Figure S2: XRD patterns (a), Raman spectra (b), measured N_2 adsorption-desorption isotherm (c), UV-Vis absorbance spectra (d), and Tauc plot for band gap energy determination (e) for the TiO_2 photocatalyst P25.

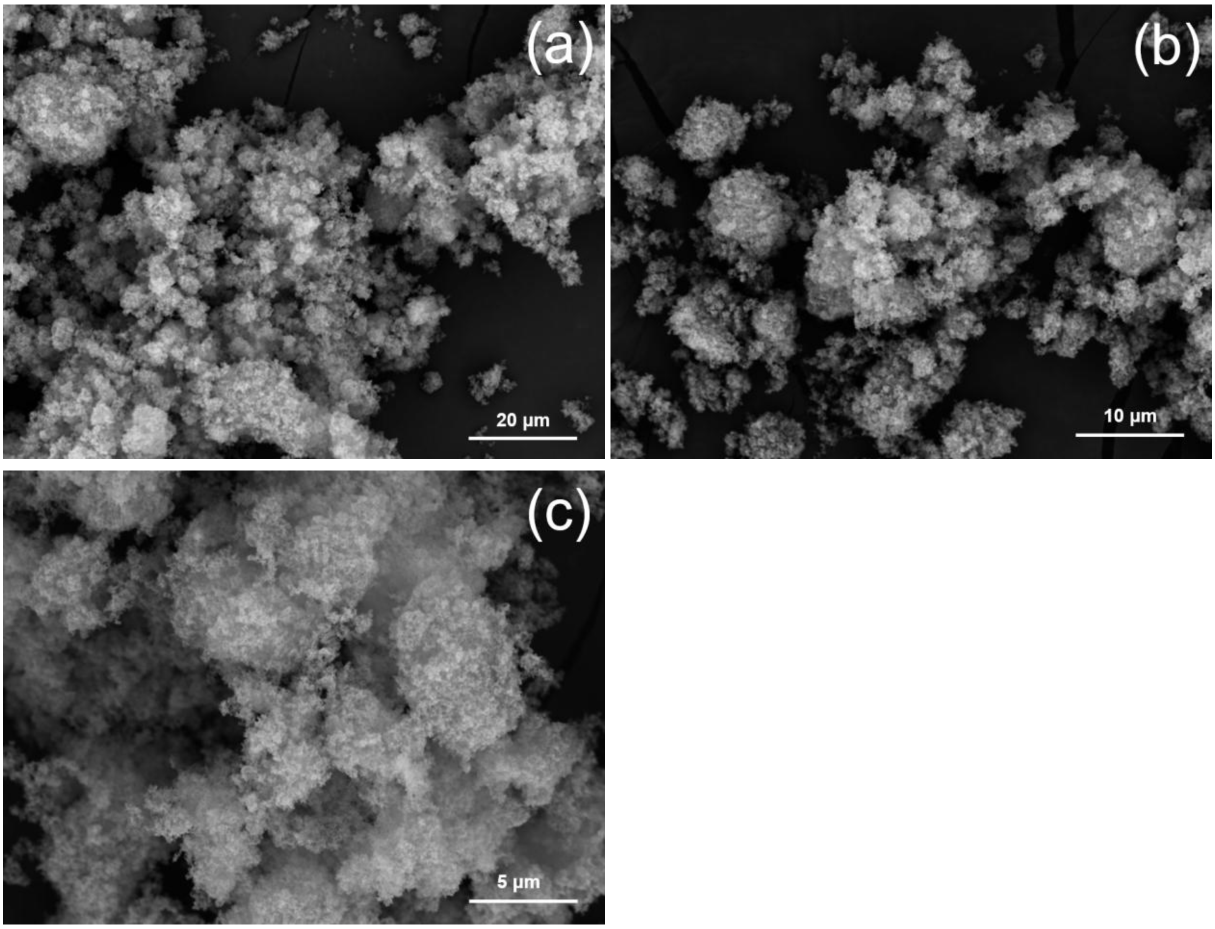

Figure S3: SEM images of the commercially available TiO_2 photocatalyst P25 acquired at different magnifications: (a) 20 µm, (b) 10 µm, and (c) 5 µm.

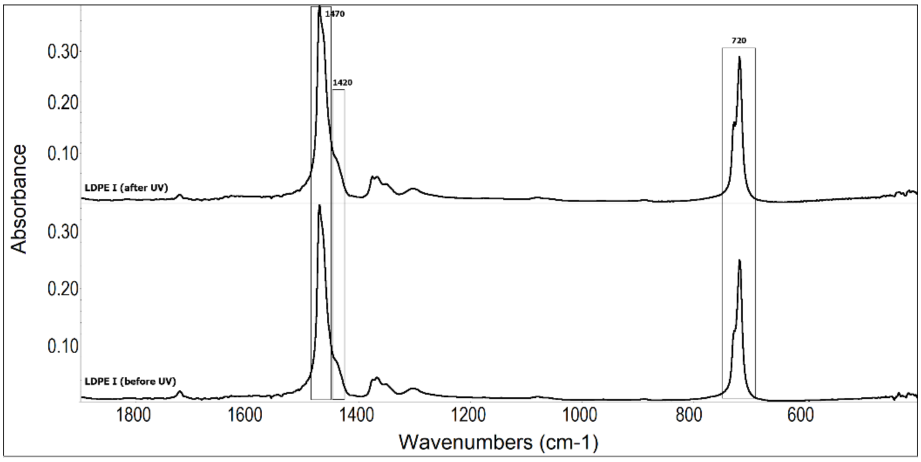

Figure S4: IR spectra of bulk samples of LDPE I before UV and after UV exposure. The identification of IR bands according to Figure 1.

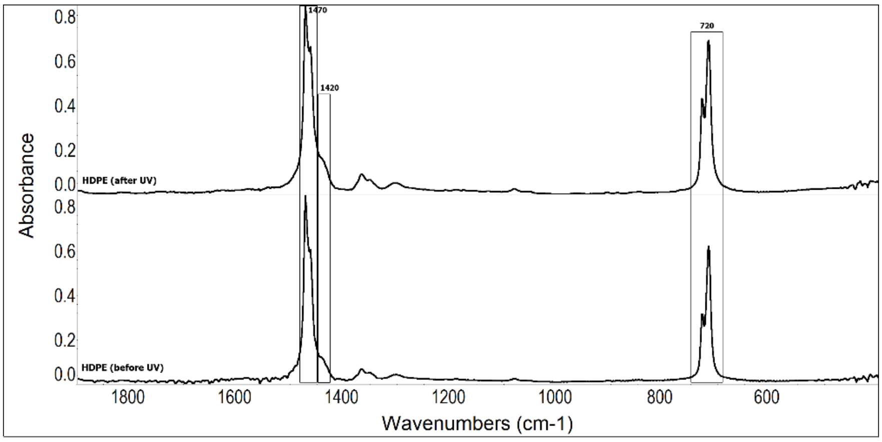

Figure S5: IR spectra of bulk samples of HDPE before UV and after UV exposure. The identification of IR bands according to Figure 1.

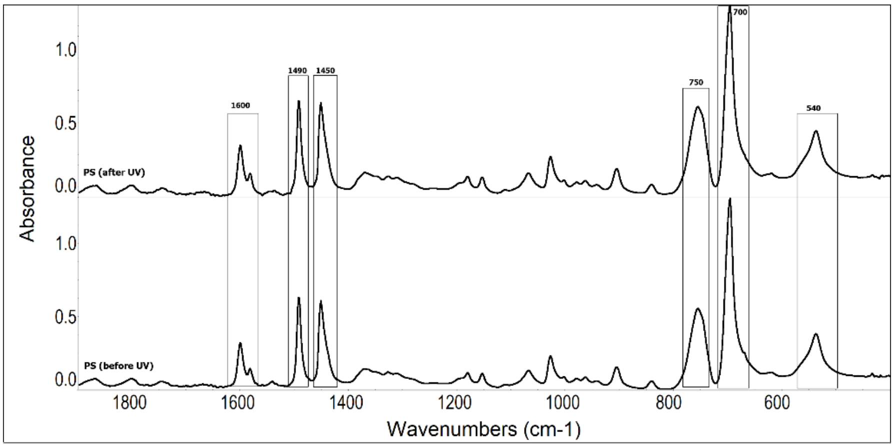

Figure S6: IR spectra of bulk samples of PS before UV and after UV exposure. The identification of IR bands: 1600 cm^{-1} – C=C stretching (in aromatic circle), 490 cm^{-1} – C=C stretching (in aromatic circle), 1450 cm^{-1} – C-H bending (in aliphatic chain), 750 cm^{-1} – C-H out of plane bending (in aromatic circle), 700 cm^{-1} – C-H out of plane bending (in aromatic circle), 540 cm^{-1} – C=C bending (skeletal vibration of aromatic circle).

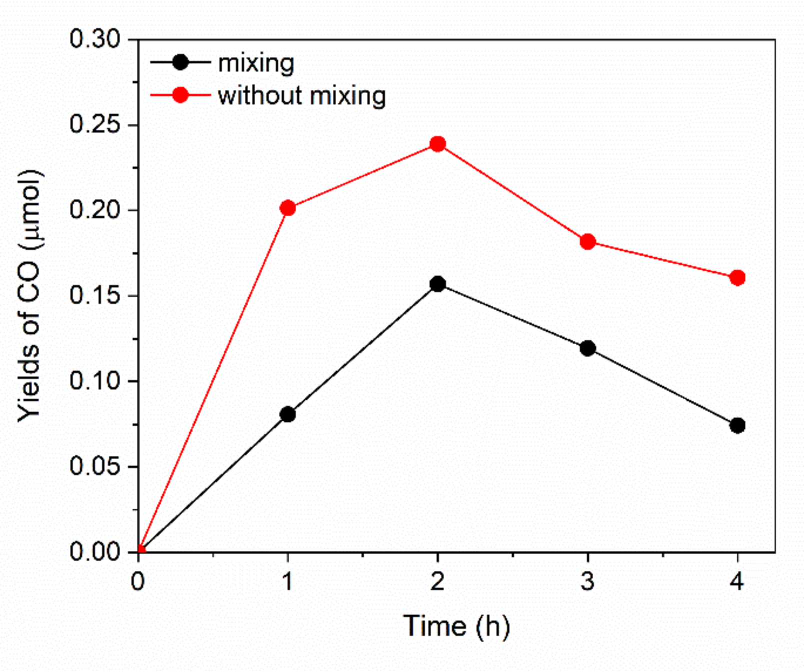

Figure S7: Generation CO during photolysis in presence of LDPEII in time with or without mixing.

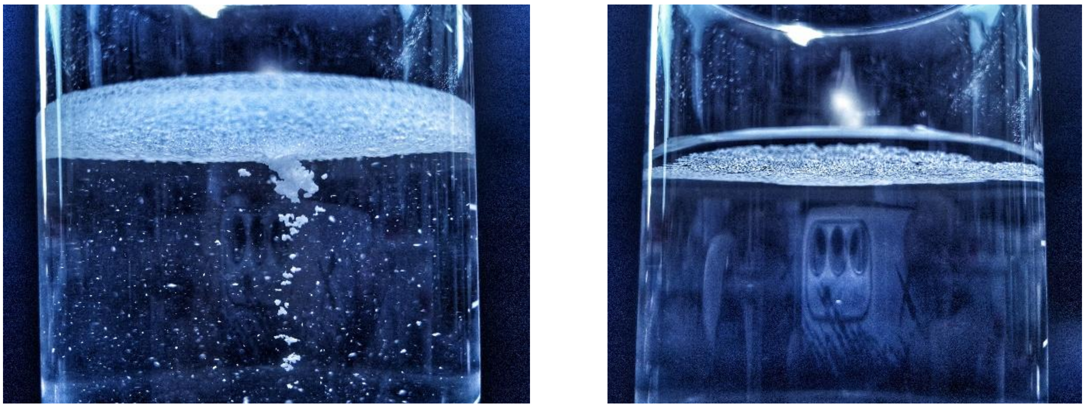

Figure S8: Photographs of reaction suspensions demonstrating differences in LDPE II dispersion under stirred and non-stirred conditions.

Table S1: Product yields obtained after 4 hours of irradiation by photocatalysis and photolysis.

	**Photolysis**			**Photocatalysis**		
Yields of products/samples	**H_2 (μmol)**	**CH_4 (μmol)**	**CO (μmol)**	**H_2 (μmol)**	**CH_4 (μmol)**	**CO (μmol)**
PS	3.03	0.63	0.04	4.60	0.67	0.04
HDPE	4.33	0.52	0.04	2.49	0.41	0.02
LDPE I	4.56	0.76	0.07	3.35	0.44	0.05
LDPE II	6.66	1.20	0.07	4.54	1.33	0.16

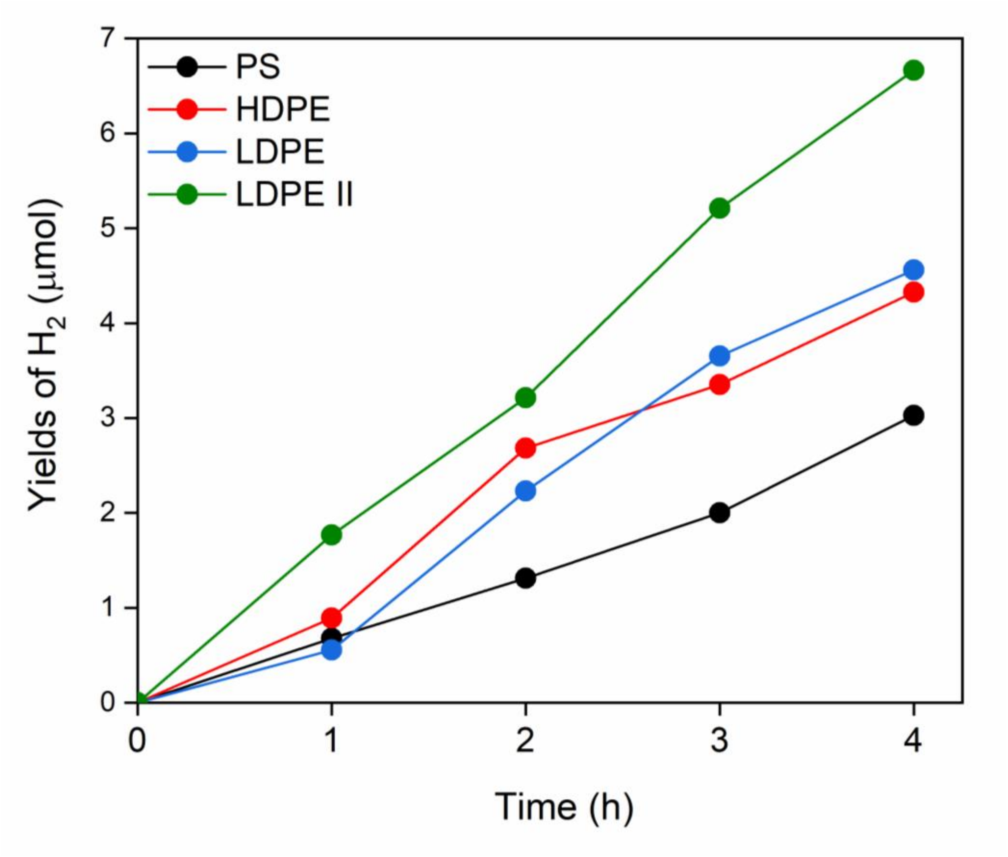

Figure S9: Generation H_2 during photolysis in presence of investigated microplastics in time.

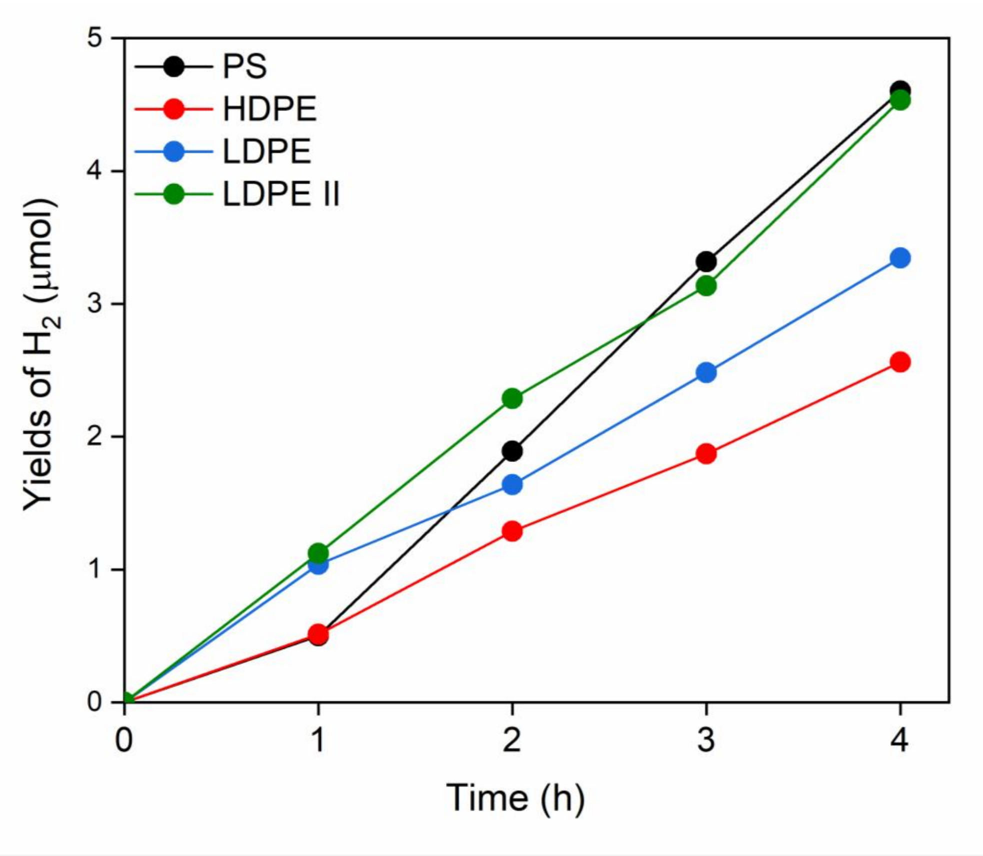

Figure S10: Generation H_2 during photocatalysis in presence of investigated microplastics in time.

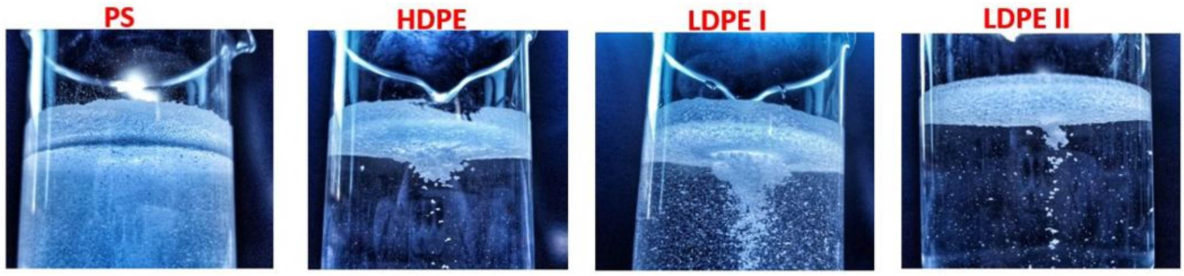

Figure S11: Photographs of reaction suspensions during photocatalytic experiments showing the different dispersion behaviour of PS, HDPE, LDPE I, and LDPE II.

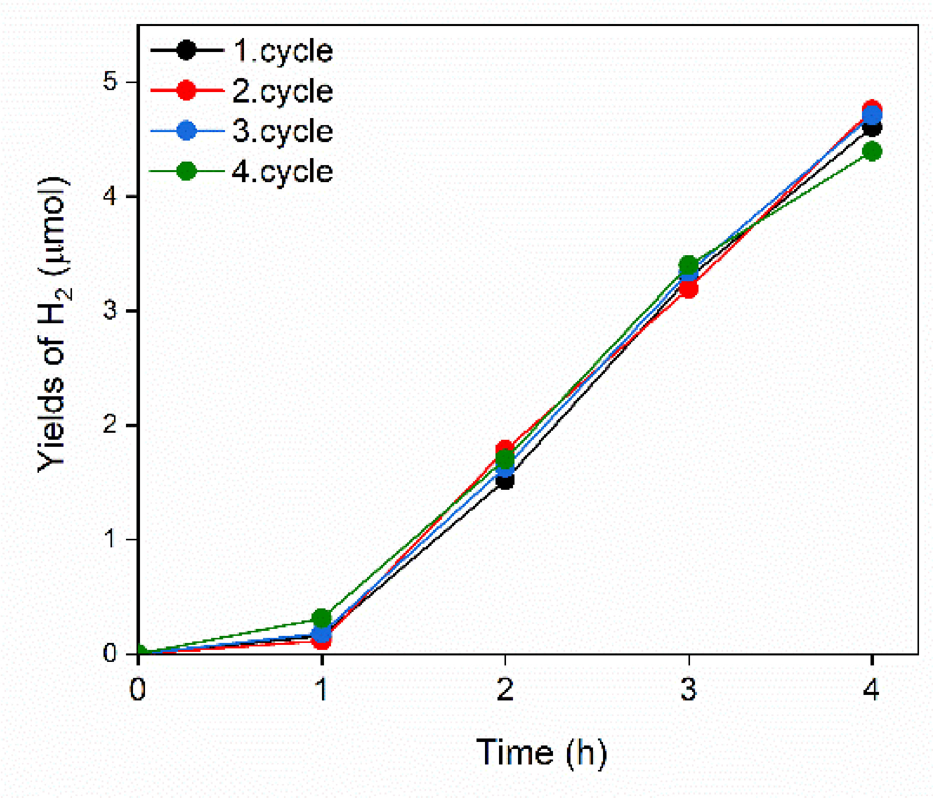

Figure S12: Stability test of PS sample during four photocatalytic measurements.
